# MicroRNAs as Biomarkers and Therapeutic Targets in Female Infertility

**DOI:** 10.3390/ijms252312979

**Published:** 2024-12-03

**Authors:** Lucía Chico-Sordo, Juan A. García-Velasco

**Affiliations:** 1 IVIRMA Global Research Alliance, IVI Foundation, Instituto de Investigación Sanitaria La Fe (IIS La Fe), 46026 Valencia, Spain; lucia.chico@ivirma.com; 2 IVIRMA Global Research Alliance, IVIRMA Madrid, 28023 Madrid, Spain; 3 School of Health Sciences, Medical Specialties and Public Health, Obstetrics and Gynecology Area, Rey Juan Carlos University Alcorcón, 28922 Madrid, Spain

**Keywords:** infertility, microRNA, ovarian alterations, uterine disorders

## Abstract

The study of microRNAs (miRNAs) has emerged in recent decades as a key approach to understanding the pathophysiology of many diseases, exploring their potential role as biomarkers, and testing their use as future treatments. Not only have neurological, cardiovascular diseases, or cancer benefited from this research but also infertility. Female infertility, as a disease, involves alterations at multiple levels, such as ovarian and uterine alterations. This review compiles the latest studies published in humans that link female disorders that affect fertility with altered miRNA profiles. Studies on ovarian alterations, including diminished ovarian reserve (DOR), poor ovarian response to stimulation (POR), premature ovarian insufficiency (POI), and polycystic ovary syndrome (PCOS), are summarized and classified based on the expression and type of sample analyzed. Regarding uterine disorders, this review highlights upregulated and downregulated miRNAs primarily identified as biomarkers for endometriosis, adenomyosis, decreased endometrial receptivity, and implantation failure. However, despite the large number of studies in this field, the same limitations that reduce reproducibility are often observed. Therefore, at the end of this review, the main limitations of this type of study are described, as well as specific precautions or safety measures that should be considered when handling miRNAs.

## 1. Introduction

Infertility is defined as the inability to achieve pregnancy after having regular unprotected sexual intercourse for 1 year. This condition affects millions worldwide, with a lifetime prevalence of 17.5% and 12.6% during the 12 months [1]. The origin of infertility can be in both male and female factors, with approximately 50% of the cases attributed to men [2]. Congenital bilateral absence of the vas deferens associated with mutations of the cystic fibrosis gene is one of the primary genetic origins [3], followed by varicocele as an acquired factor [4], and in another percentage of cases, the origin remains unknown. Focusing on women, infertility can be associated with endocrine [5], ovarian, or uterine alterations. However, in many cases, as in men, it is not possible to determine the infertility origin. Ultimately, conception and pregnancy take place in the woman, and disturbances at any of these levels may result in failure of conception or even miscarriage.

In many instances, assisted reproductive technologies (ARTs) are used to overcome infertility and conceive [6,7]. Establishing an accurate early diagnosis is crucial during this process. The origin of infertility and the available treatments will determine the prognosis when the infertility diagnosis is determined. For this reason, much of the research efforts in the field of ARTs are focused on (1) facilitating and accelerating the diagnosis of infertility, (2) providing a realistic prognosis, and (3) developing new therapies for this condition. In recent years, miRNAs have become promising molecules that could help achieve any of the above mentioned objectives. Although this field of study is still in its very early stages, several researchers have explored the potential of miRNAs from different diseases or conditions. Given the invasiveness of diagnostic techniques, such as laparoscopy [8] or hysterectomy [9], used in women, many studies analyzing the role of miRNAs at the reproductive level focus on women, hence this literature review. However, despite a large number of published researches, specific considerations and precautions must be considered when dealing with miRNAs.

This literature review article summarizes the published preliminary studies in humans that postulate miRNAs as tools for the diagnosis, prognosis, and treatment of female reproductive disorders.

## 2. microRNAs

### 2.1. Identification and Biogenesis

Although not translated into proteins, some RNA molecules are functional and called non-coding RNA (ncRNA) [10]. These types of RNAs are classified according to their size. If it is more significant than 200 nucleotides, they are considered long non-coding RNAs (lncRNAs), while if it is smaller, they are classified as small non-coding RNAs (sncRNAs). Multiple types of sncRNAs exist as miRNAs or Piwi-interacting RNAs (piRNAs) [11]. Still, this review will focus on miRNAs because they are widely conserved molecules across species, and a single miRNA can exert its regulation on many different targets. They are RNA molecules of approximately 22 nucleotides that play a crucial role in regulating gene expression at the post-transcriptional level [12]. Its discovery dates back to 1993 in the organism *Caenorhabditis elegans* (*C. elegans*), when Lee et al. [13] demonstrated the existence of the *LIN-4* gene, which, after transcription, did not code for a protein but gave rise to two small RNA molecules. These small RNAs were complementary to the *LIN-14* gene mRNA in the 3′ untranslated region (UTR), and as a result of the interaction, a decrease in Lin-14 protein levels was observed [14]. Years later, Reinhart et al. (2000) [15], working on the same model organism, would reach the same conclusion by discovering a new *lethal-7* (*LET-7*) gene that showed a similar pattern.

**Figure 1:**
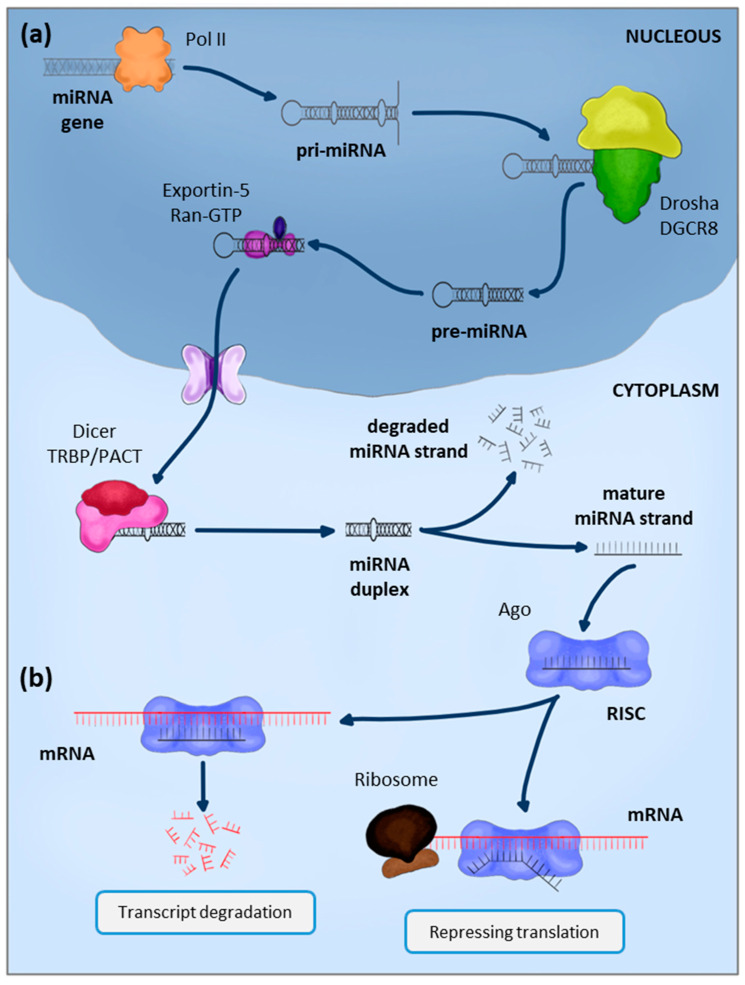
Biogenesis of miRNAs and their regulatory function of gene expression. (**a**) miRNAs obtention and maturation process. In the canonical pathway, RNA-polymerase II transcribes the miRNA gene; the initial transcript generated is called pri-miRNA. Then, thanks to the type III ribonuclease activity of Drosha, the double-stranded RNA will be recognized and removed from the poly-A tail, giving rise to the pre-miRNA. This pre-miRNA is exported from the cell nucleus to the cytoplasm bound to Exportin-5 via the RanGTP/RanGDP transport system. Once in the cytoplasm, another type III ribonuclease, Dicer, cleaves the stem-loop-terminal region and generates a double-stranded miRNA. One of the two strands that form the mature miRNA will be degraded (called the passenger strand) by the argonaute protein with endonuclease activity or RNases present in the cytoplasm. The other strand of the miRNA will remain bound to the argonaute protein to perform its function. (**b**) Gene silencing. Post-transcriptional regulation of the expression of different genes can occur in two ways. (**Left**) When the complementarity of bases between the miRNA and the target mRNA is total, the argonaute protein itself, with its endonuclease activity, will destroy the miRNA. (**Right**) However, if this complementarity is not total, the RISC complex will remain and impede the progress of the translation process.

Currently, multiple miRNAs have been identified, and their processing has been described in detail [16,17,18]. Although there are different post-transcriptional processing pathways, the majority of conserved miRNAs in all vertebrates originate from the canonical pathway explained below (Figure 1a) [19]. In general terms, RNA polymerase II (Pol II) transcribes the miRNA genes, generating a primary miRNA transcript (pri-miRNA) in the cell nucleus [20]. This pri-miRNA varies in size from hundreds of nucleotides to tens of kilobases and forms a stem-loop structure in which a hairpin and a base can be distinguished. The pri-miRNA is characterized by being polyadenylated and capped, and it can be spliced [21]. In the nucleus, it will undergo an initial processing by which the base part is cleaved, leaving only the hairpin miRNA precursor (pre-miRNA) of about 70 nucleotides. The machinery in charge of this first step in miRNA processing is the microprocessor complex formed by the RNase III enzyme Drosha and the double-stranded RNA-binding protein DiGeorge syndrome critical region gene 8 (DGCR8) [22]. Then, thanks to Exportin-5 [23,24,25], the pre-miRNAs will be transferred to the cell cytoplasm, where the next Dicer-mediated processing step will occur. Dicer is an RNase III enzyme that, after association with double-stranded RNA (dsRNA)-binding proteins, will cleave the stem-loop terminal region of pre-miRNA, generating a miRNA duplex. This duplex consists of a double strand of approximately 22 nucleotides. The dsRNA-binding proteins in human cells can be PKR activator protein (PACT) or transactivation response RNA-binding protein (TRBP) [26]. From the generated duplex, one of the strands will be bound to argonaute proteins (Ago) [27], the RNA-induced silencing complex (RISC) will be formed, and the other strand will be degraded [28].

By the process detailed above, canonical miRNAs are obtained. However, it is important to note the existence of miRNA isoforms called isomiRs. These small RNA molecules differ from the canonical miRNAs in length, sequence, or both and sometimes even have a different function [29,30]. Their synthesis takes place using the same process as canonical miRNA synthesis. Alterations in the process, such as imprecise Drosha or Dicer excision, addition or deletion of nucleotides at the 5′ or 3′ ends, or inclusion of single nucleotide polymorphisms (SNPs), are the specific origin of isomiRs [31]. Therefore, their presence should be taken into account in studies on miRNAs as they may be missed in miRNA detection and expression methods [32].

### 2.2. Role in Biological Processes

The early findings about miRNAs mainly were attributed to the organism *C. elegans,* and these findings were further supported by research on the regulatory function of these molecules in *Drosophila* [33,34]. The primary function of miRNAs is to regulate gene expression by post-transcriptional gene silencing acting on messenger RNA (mRNA) [35,36]. Specifically, this is the process of RNA interference, a biological mechanism mainly mediated by small RNA molecules such as miRNAs and small interfering RNAs (siRNAs). For miRNA-mediated interference to occur, the miRNA, as a part of the RISC complex, must travel to its target mRNA and, through base complementarity, carry out its function (Figure 1b). Two regions are essential for the miRNA binding to target mRNA: the miRNA seed region and the 3′-UTR region of the mRNA. The miRNA seed region is between nucleotides 2 and 8 of the miRNA 5′ end [37]. The 3′-UTR region is an untranslated region of the mRNA localized at the 3′ end, also called the trailer sequence [38]. Total or partial base complementarity between the miRNA seed region and 3′-UTR mRNA can be used to characterize the phenomenon as translational repression or mRNA degradation [39,40]. In the first case, the direct and immediate degradation of mRNA will be induced and performed by Ago endonuclease activity [41,42]. If the base complementarity is partial, translation inhibition will be indirect since the RISC complex will stay attached to the mRNA, forming a spatial barrier that will stop the translation machinery from progressing [43].

Mature miRNAs mostly work in the cytoplasm of cells. However, recent studies have shown that these molecules can also act at the nuclear level [44]. Although the negative regulation of complementary target mRNAs is the main function at the cytosolic level, there are several ways in which this regulation can be executed at the nuclear level. Among these new approaches to miRNA-mediated regulation are the interaction between miRNAs and other ncRNAs as circular RNAs (circRNAs) [45,46] and the silencing of genes coding for proteins involved in the maturation process of the miRNAs themselves [47] or even indirectly involved in epigenetic modifications [48].

### 2.3. miRNA Availability

As explained above, miRNAs are synthesized in the cell nucleus and exported to the cytoplasm to complete their maturation process. Once the miRNAs have matured, they will mainly exert their function in the cytoplasm. However, it is important to mention that many miRNAs migrate out of the cell, having an important role as chemical messengers [49,50]. As a consequence of this migration, it is possible to identify miRNAs in body fluids such as blood [51], urine [52], saliva [53], follicular fluid [54], seminal fluid [55], and breast milk [56], among others. Furthermore, it has been observed that despite their ribonucleic acid nature, they are characterized by high stability in biofluids [57]. The reason is that miRNAs are not released in isolation; instead, they move and form vesicles or ribonucleoprotein complexes. Thanks to these bindings, the RNases present in biofluids cannot degrade them. Specifically, 90% of circulating miRNAs are bound to proteins such as AGO2 [58], GW182 [59], and HDL [51], while the remaining 10% travel inside exosomes or microvesicles [57,60].

### 2.4. miRNAs as a New Approach in Diagnosis and Treatment

After the last few years of study on miRNAs, it has been confirmed that these molecules play a fundamental role in the appearance or progression of diseases. While providing information on the molecular processes mediated by miRNAs that may be altered in different conditions, all these studies also represent a major advance for diagnosing and treating diseases. One of the most promising roles for miRNAs is their use as biomarkers. Different researchers have explored these molecules in cancer [61,62,63,64], immunological [65] and neurological disorders [66,67,68], or even cardiovascular diseases [69,70,71]. Recently, miRNAs have also been evaluated as hopeful therapies using an analog or mimic [72] and inhibitors or antagonists [73] of the miRNA in question.

## 3. miRNAs and Infertility

The World Health Organization (WHO) classifies infertility as a disease [74]. Therefore, like many other pathologies mentioned above, miRNAs can also play a role in the diagnosis and treatment of infertility. However, infertility is a complex condition that involves the interaction of biological [75,76,77], emotional [78,79], anatomical or physical [80,81,82,83,84], and social factors [85,86,87]. In addition, infertility can have its origin in either the man or the woman. Specifically, infertility in women can be due to ovulatory disorders and uterine alterations, among others, as hormonal problems. The vast majority of published research on miRNAs and female infertility works with ovarian and endometrial samples, subsequently analyzing whether the miRNA expression pattern found in that type of sample is reflected in biofluids such as blood. For this reason, the latest research on miRNAs related to ovarian and uterine disorders will be discussed below.

### 3.1. Ovarian Alterations

One of the causes of female infertility is ovarian insufficiency. This concept includes poor ovarian response, premature ovarian insufficiency (also called premature ovarian failure, POF), and advanced maternal age. All these are characterized by reduced ovarian reserve, but the boundaries between these concepts are poorly defined [88]. Consequently, in many cases, the diagnosis is not clear since clinical features may overlap. This problem is directly reflected in the studies that analyze the relationship between miRNAs and the different situations of ovarian failure.

The following is a summary of research that has looked into the function and potential of miRNAs in cases of POI, POR, and DOR. Furthermore, a detailed discussion of PCOS, another extremely common ovarian condition, will be provided. Table 1 summarizes the miRNAs found to be altered in each ovarian disorder analyzed. They are classified by color depending on the nature of the sample studied.

#### 3.1.1. Diminished Ovarian Reserve 

Currently, there is no single accepted definition for the term DOR [115]. At the clinical level, it is mainly defined as a reduction in ovarian follicle number, a low ovarian response to stimulation, and a poorer oocyte quality [88]. Therefore, one of the key concepts is ovarian reserve, which can be assessed by the antral follicle count (AFC) or anti-müllerian hormone (AMH) level [116,117]. In most studies that examined the expression of miRNAs in DOR cases, the AMH and AFC were shown to differ between the DOR group and the control group. However, these differences in ovarian reserve markers are accompanied by statistically significant differences in the mean age of the groups. Although the concept of DOR only refers to a woman’s ovarian reserve, it is important to mention that it is a normal physiological process linked to age and is very common in women in their mid-40s. However, when it appears in young women, it is a pathological condition [115].

An analysis of miRNAs in this patient profile has been conducted to comprehend the pathophysiology and physiology of DOR. Granulosa cells (GCs) and cumulus cells (CCs), which are ovarian follicle cells, have been the primary subjects of this kind of study. The main reason is that the decrease in oocyte competence in women with DOR could originate from abnormal regulation by the cells accompanying the oocyte in the environment of the antral follicle. The miRNAs miR-221-3p, which targets the gene *FOXO1* [109], and miR-106a, whose target is *ASK1* [110], showed reduced levels in DOR patients. In both studies, alteration in these miRNAs led to increased GCs or CCs apoptosis. The study by Woo et al. [89] also evidenced lower levels of miR-16-5p, triggering an increase in *MAPK* and *WNT3*, promoting elevated cell proliferation, differentiation, and apoptosis. The same study demonstrated higher levels of miR-128-3p, causing a decrease in *TGFBR1*, an alteration previously observed in older women. Consequently, the DOR profile would be similar to older women’s profiles. MiR-6881-3p [90] and miR-484 [91], also with higher expression in DOR women, caused decreases in *SMAD4* and *YAP1*, respectively, increasing the levels of apoptosis of GCs. However, not only has the miRNA profile been studied in cells directly related to folliculogenesis, but miRNAs have also been evaluated in follicular fluid (FF). Since one of the primary roles of exosomes is cell-to-cell communication, the amounts of various miRNAs found in FF exosomes have been specifically examined. Among the most recent studies, Shen et al. [93] found a lower expression of miR-122-5p, miR-1246, and miR-130b-3p. The miRNAs miR-342-3p, miR-483-3p, and miR-625-3p were the most abundant FF exosomes from DOR patients. The fact that there were no statistically significant variations in the mean age of the DOR groups and the control group with normal ovarian reserve is a good aspect of this study. This fact cannot be stated in another study, the one carried out by Xie et al. [94], where despite finding downregulated miR-21-5p and upregulated miR-28-3p, miR-155-5p, and miR-29a-5p, the mean age between the study groups was statistically significant.

Each of the aforementioned research studies, which sought to elucidate the role of miRNAs in the etiology of DOR, proposes miRNAs as potential biomarkers for this condition. miRNAs as biomarkers would be an additional parameter, which, together with AMH and AFC values, would allow professionals to narrow down and diagnose this condition accurately. However, specific studies have focused on analyzing female blood samples with DOR, searching for non-invasive or minimally invasive biomarker candidate miRNAs. MiR-106a [110] and miR-4463 [92], the first decreased and the second increased in DOR patients, have been tested simultaneously in serum and follicular cell samples. The research by Abu-Halima et al. [118] did not focus on circulating miRNAs in serum or plasma but instead profiled miRNAs present in blood cells from women with lower AMH, normal AMH, and higher AMH.

#### 3.1.2. Poor Ovarian Response 

POR refers to a low number of competent oocytes retrieved after ovarian stimulation. This type of response to ovarian stimulation is mainly associated with maternal age over 40 years; it is typical in women with a previous history of cycle cancellation due to a low number of oocytes retrieved and a DOR [119,120].

Compared to research about DOR, fewer publications examine the expression profile of miRNAs in women with POR. However, many of the studies performed in DOR are executed during in vitro fertilization (IVF) treatments after ovarian stimulation. Once stimulation has been performed and oocytes are collected, GCs, CCs, or FF obtained during oocyte pick-up are used for miRNA analysis. Therefore, ovarian stimulation can influence the miRNA signature, and considering that DOR and POR conditions often overlap, it is difficult to determine whether these types of studies are exclusive to women with a DOR profile. The studies on POR women have been performed with the same objective as studies on DOR: to analyze the role of miRNAs in the etiology of this condition. These investigations have also been conducted in GCs, where an increase in miR-23a was evidenced. This is linked to an elevation of apoptosis in GCs, which results in follicular atresia by inhibiting *SIRT1*. The increase in apoptosis was evidenced by elevated Caspase-3 [95]. In CCs, the miRNA that recorded elevated levels in POR women was miR-21-5p, demonstrating independence concerning low estradiol levels [96]. The study by Zhang et al. [97] focused on FF, differentiated between younger and older POR women, and only in the younger group were high levels of miR-15a-5p detected. This upregulated miRNA promoted cell apoptosis by reducing *BCL2* levels.

Although studies on miRNAs that may play a role in the cause of POR are promising, to date, none have been explored as a treatment or biomarker per se. Currently, the clinical approach to POR cases is focused on obtaining a greater number of competent oocytes. To this end, smaller follicles, whose oocytes may also have compromised nuclear competence, are often aspirated during oocyte collection. From the point of view of miRNAs, and for clinical reassurance, there were no significant differences in the miRNA signature associated with oocyte maturation (miR-451 and miR-574) in FF from both small and large follicles [121]. The study showed that this strategy versus the POR condition does not imply a lower nuclear competence of the oocyte aspirated from the smaller follicles.

#### 3.1.3. Premature Ovarian Insufficiency 

As previously described, a loss in ovarian function is typical of advanced maternal age. Still, when it occurs before the age of 40, it refers to the term POI, also known as POF [122]. Clinically, the diagnosis is made when a woman presents menstrual irregularities, an increase in follicle-stimulating hormone (FSH), and low-level estrogen [123]. This alteration is represented in approximately 1% of the female population [124]. The main consequence of this ovarian activity decline is infertility [125]. Nonetheless, there are other consequences that negatively impact women’s health, usually associated with menopause. Osteoporosis [126], cardiovascular disease [127], and sexual dysfunction [128], all of them linked to a decrease in estrogen production, accompany the symptomatology of POI. In addition, the consequences on the psychological well-being of these women, like emotional distress, are devastating [129,130,131].

Respecting the etiology of POI, genetic causes [132,133], immunological alterations [134,135], metabolic disorders [136], and environmental toxins [137] have been identified. The induced POI by different agents as chemotherapeutic in animal models has made it possible to profile the miRNAs involved in this condition [138,139,140,141,142]. However, human studies are very difficult to carry out because follicular aspiration is not performed in many of these women due to cycle cancellation. After the cancellation of oocyte retrieval, collecting FF, GC, or CC samples for research is impossible. For this reason, many of the studies about POI are performed on blood. Recent plasma studies have studied miRNAs and the role of other ncRNAs, such as circRNA. They act as “molecular sponges”, binding to and absorbing miRNAs and indirectly controlling the expression and function of the target mRNA [143]. A lower expression in circRNA_008901 and circRNA_403959 was observed in POI patients, suggesting these as biomarkers for this condition [144]. Other studies, following a more traditional experimental design, have focused on the direct search for miRNAs with altered expression in the blood of women with POI. These investigations have evidenced the importance of downregulated miR-22-3p [111] or the overexpression of miR-23a and miR-27a in women suffering from this condition [98]. Thanks to these types of studies that have suggested potential miRNAs that could directly affect the cause of POI, it has been possible to transfer the investigations from blood samples to cell cultures. The group of Nie et al. (2015) [145], based on their previous studies from blood samples [98], transfected primary cultures of GCs obtained from patients undergoing IVF techniques with miR-23a and miR-27a. Overexpression of these miRNAs showed that their increase caused a decrease in *SMAD5*, promoting apoptosis in human GCs. Studies with established cell lines, such as KGN cells (human granulosa-like tumor cells), complement those carried out with primary cultures. Co-transfection of these cells with miR-146b-5p and the lncRNA DLEU1 showed that there is indeed an interaction between the two so that DLEU1 promotes cell apoptosis by reducing the inhibitory effect of miRNA on cell apoptosis [146]. Not only are granulosa cells the direct target of these studies, but the clinical complexity of POI cases has led to the exploration of other cell types, such as Th17 [147]. These cells are a type of lymphocytes that activate the immune system, causing inflammation, whose activation is mediated by miR-326. The results recorded an increase in this miRNA in Th17 cells from patients with POF.

Previously, clinical management of POI cases was often limited to oocyte donation to increase pregnancy success rates. However, many new clinical approaches and promising therapies are now being implemented in this patient profile [148]. These new therapies also represent a promising front of study for miRNAs; profiling the differentially expressed miRNAs in POI patients under these treatments could provide important information on their role in the POI origin.

#### 3.1.4. Polycystic Ovarian Syndrome 

PCOS affects 4–20% of pre-menopausal women worldwide and is characterized by ovarian dysfunction, polycystic ovaries, and hyperandrogenism. Women with PCOS have reduced fertility [149]. However, this syndrome also involves other alterations, especially at the endocrine and metabolic levels [150]. These characteristics and manifestations include hirsutism, acne, obesity, and insulin resistance [151]. This variety in the alterations associated with PCOS complicates the study of the miRNAs differentially expressed in these patients since there is significant heterogeneity between studies [152].

MiRNAs in women with this profile have been investigated in blood samples, FF, GCs, and CCs. In blood, published studies have measured miRNA levels in both serum and plasma. With respect to serum studies, miR-222, miR-146a, miR-30c [105], miR-21-5p, miR-23a-3p, and miR-26a-5p [107] were upregulated in PCOS women, whereas miR-155 [112], miR-103-3p [107,113], miR-376a-3p [107,113], miR-19b-3p, miR-222-3p [107], miR-139-5p, miR-28-3p [113], and miR-320 [114] were downregulated. In plasma, miR-93, miR-223 [104], miR-126-3p, miR-146a-5p [106], miR-151a-5p, and miR-4488 [108] were identified with higher expression in women with PCOS, whereas the levels of miR-20b-5p, miR-106a-5p, miR-18a-3p [106], and miR-223-3p [108] were lower. Among the studies on the expression profile of miRNAs in the FF of PCOS women, miR-539-5p [102], miR-650, and miR-663b [103] evidenced downregulation. MiR-3131, miR-206, miR-204-5p, miR-100-5p, miR-193a-5p [102], miR-381-3p, miR-199b-5p, miR-93-3p, miR-361-3p, miR-127-3p, miR-1238-3p, miR-382-5p, and miR-425-3p [103] recorded elevated levels in PCOS patients. One of the studies worked with FF and CCs found in both types of samples, and the same miRNAs were increased (miR-212-3p, miR-490-5p, and miR-4643) and downregulated (miR-647) [101]. Regarding GCs, Li et al. (2019) [99] determined by quantitative polymerase chain reaction (qPCR) that miR-33b and miR-142 expression was upregulated, whereas miR-423 was downregulated. However, in the study by Xu et al. (2023) [100], they went a step further, and after showing an increase in miR-1298-5p in GCs from PCOS patients, they transferred their study to cultured COV343 human ovarian granulosa cells to analyze their molecular role in this condition.

Despite the large number of studies on miRNAs and PCOS, the conclusions gained are conditioned by certain inherent characteristics of this condition. For this reason, it is necessary to define the PCOS study population clearly.

#### 3.1.5. Overlapping miRNAs in Ovarian Alterations

After the analysis of the different miRNAs with differential expression in each ovarian disorder exposed, it can be observed that many of them have been identified in different alterations or even multiple times in the same disorder. MiR-106a was identified by its low expression in both blood and follicular cells in women with DOR [110]; its expression was also lower in PCOS blood, although in the study, it was specified that the miRNA identified was miR-106a-5p [106]. Something similar occurred with miR-155 [112] and miR-28-3p [113] downregulated in blood from PCOS women and upregulated in follicular fluid from DOR [94], although specifically, the first one was miR-155-5p [94]. Another miRNA with differential expression between DOR and PCOS was miR-21-5p; this miRNA recorded low expression in the FF of DOR [94] and high expression in the blood of PCOS [107]. In addition, this miRNA was upregulated in follicular ovarian cells of POR women [96]. The miRNA miR-23a showed elevated levels in POR women (in follicular cells) [95] and in the blood of POI [98] and PCOS cases; in the latter, it was miR-23a-3p [107].

The results within the group of women with PCOS are highly controversial. MiR-222 [105] and miR-223 [104] were upregulated in blood samples, whereas the specific miRNAs miR-222-3p [107] and miR-223-3p [108] registered low levels in the blood of these patients. Finally, both miR-146a [105] and miR-93 [104] were notable for their higher expression in blood samples from women with PCOS, although in the first case, both miR-146a and miR-146a-5p [106] were detected, and in the second case, it was miR-93-3p [103] that was also upregulated in the follicular fluid from these patients.

### 3.2. Uterine Alterations

Like the ovary, the uterus is an organ of great importance for fertility in the female reproductive system. This organ is formed by three layers, which, from the inside to the outside, are the endometrium, myometrium, and serosa [153]. Specifically, the endometrium is the most important one since its thickness varies cyclically in response to sex hormones during the menstrual cycle. Because the embryo will implant in the endometrium and the pregnancy will continue, the primary function of this tissue is associated with pregnancy. As in other tissues, miRNAs also have a regulatory role [154,155]. The endometrial miRNA profile can vary depending on the menstrual cycle phase [156,157], endometrial receptivity for embryo implantation [158,159,160], or in situations of implantation failure [161,162,163]. In addition, this profile may be altered by disorders closely linked to fertility, such as endometriosis or adenomyosis [164]. The miRNAs that were discovered to be changed in endometriosis and adenomyosis are listed in Table 2. Depending on the type of sample examined, the miRNAs are categorized by color.

#### 3.2.1. Endometriosis

Endometriosis is a disease characterized by endometrial tissue outside the uterine cavity. It is an estrogen-dependent chronic inflammatory disease [182,183]. It is estimated that endometriosis affects 10% of women of reproductive age [184]. Its symptoms include pelvic pain, menstrual irregularities, heavy bleeding, and pain during urination and sexual intercourse [185]. However, the symptomatology is somewhat generic, resulting in a 6–10 year delay in diagnosis. The reference technique for diagnosing this condition is laparoscopy, characterized by high diagnostic sensitivity and specificity. Invasiveness and dependence on the surgeon’s judgment for identifying lesions are intrinsic to this technique [186]. This is why multiple studies have been carried out to explore the capacity of miRNAs as diagnostic biomarkers of endometriosis [187].

Numerous studies have been aimed at finding differentially expressed miRNAs in endometriosis. The latest publications work with samples obtained non-invasively, such as saliva [188]. One of the most explored samples in endometriosis is blood, obtained through a minimally invasive process. Studies on blood plasma, such as that by Papari et al. (2020) [176], showed low levels of miR-199a-3p, miR-143-3p, miR-340-5p, let-7b-5p, miR-21-5p, miR-103a-3p, miR-17-5p, and miR-20a-5p. These last two miRNAs were also detected as downregulated in women with endometriosis in the study by Jia et al. (2013) along with miR-22 [178]. However, in both studies, no differences were established regarding the stage of endometriosis severity, while Bashti et al. (2018) considered this criterion [167]. Among the miRNAs the last study detected as differentially expressed in women with endometriosis, miR-31 stands out for its lower expression and miR-145 for its higher expression. In blood serum samples, miR-122, miR-199a [168], miR-125b-5p, miR-150-5p, miR-342-3p, miR-451a [169], and miR-30c-5p [170] registered higher levels in samples from women with endometriosis. On the contrary, miR-135a [177], miR-3613-5p [169], and let-7b [169,177] showed lower levels.

Studies carried out directly with endometrial tissue samples showed that the levels of the same miRNA can vary depending on the location of the endometrial tissue [165,174,189]. This implies the importance of the type of sample in which the analysis is performed, where even the peritoneal fluid from this patient profile is of interest [190,191]. Among the miRNAs that have been detected with a higher expression in endometrial tissue from women with endometriosis, miR-30a, miR-93 [165], miR-325, miR-492, miR-520e [166] stand out, while miR-143 [165], miR-203a-3p [166], miR-126-5p [174], miR-202-3p, miR-424-5p, miR-449b-3p, and miR-556-3p [175] registered lower levels.

Endometriosis and cancer share molecular characteristics because the cells involved in both pathologies can evade the apoptosis process; they are characterized by high proliferation and angiogenic potential [192]. For this reason, studies that focus on miRNA expressed in endometriosis and compare this condition with reproductive cancer would allow us to understand the differential processes between both [174].

#### 3.2.2. Adenomyosis

Adenomyosis is a benign uterine disorder characterized by the presence of ectopic endometrium in the myometrial tissue and an increase in uterine volume. Invasion of the endometrial glands and stroma into the adjacent myometrium causes hyperplasia of smooth muscle cells, leading to anatomical distortion of the uterine cavity, local inflammation, altered estrogen metabolism, and dysregulation of genes involved in maternal–embryonic crosstalk [193,194]. This condition is estimated to affect 20–30% of women of reproductive age [195], and the symptomatology is similar to endometriosis cases [196]. These percentages are based on diagnosis using transvaginal ultrasound and magnetic resonance imaging [197]. However, the image characteristics of adenomyosis can be confused with those of other uterine diseases such as endometriosis, as explained above, uterine leiomyoma, or endometrial polyps, making clinical diagnosis difficult. For this reason, the reference technique for the definitive diagnosis of adenomyosis requires histological analysis of the uterus after hysterectomy [198]. The invasive nature of this technique hinders and delays diagnosis. As a result, several research studies have concentrated on identifying the profile of miRNAs that are differently expressed in adenomyosis.

Unlike endometriosis, where the profile of differentially expressed miRNAs has been extensively studied in biofluids, in adenomyosis, the vast majority of studies were performed in endometrial samples. These studies showed the following miRNAs overexpressed in eutopic and/or ectopic endometrium from women with adenomyosis: miR-17 [171], miR-191, miR-181b [172], and miR-145 [173]. Among the differentially downregulated miRNAs expressed, miR-10b, miR-200c [172], let-7a [179], miR-30c-5p [199], and miR-183 [181] stood out. However, these miRNA expression levels can be altered after the application of a treatment, such as high-intensity focused ultrasound, as observed with miR-191-5p, whose levels decreased after treatment [200]. Other studies, such as Juarez-Barber et al. (2023) [201], instead of studying miRNAs in the entire endometrial tissue, have focused on the extracellular vesicles that secrete endometrial organoids from women with adenomyosis whose maintenance in vitro is possible.

#### 3.2.3. Receptivity Status and Implantation Failure

Both endometriosis and adenomyosis have high rates of infertility among their patients, mainly due to anatomical abnormalities present in their uterus [202]. In cases where surgery is not very aggressive, removal of the lesions allows pregnancy [203]. However, some women who do not suffer from any of these pathologies or ovarian disorders also have infertility. This may be due to problems linked to endometrial receptivity and embryo implantation failures.

For embryo implantation to take place, the moment in which the blastocyst-stage embryo attaches to the mother’s endometrium, it is necessary for the endometrium to be in the mid-secretory phase (days 20–24 of the menstrual cycle, approximately 6-10 days after ovulation). This crucial moment of the endometrium is known as the implantation window [204,205]. Once the blastocyst attaches to the luminal epithelium of the endometrium, it invades the epithelial cell layer to enter the stromal compartment, leaving the embryo completely covered by the luminal epithelium [206]. Endometrial receptivity can be predicted by transvaginal ultrasound and hormonal analysis [207]. However, establishing the miRNA expression profile during the implantation window would allow us to narrow down this optimal period. Ideally, achieving this objective with blood samples would be relevant since the endometrium would remain intact. Chen et al. (2023) [158] developed a predictive model based on the miRNA expression profile in blood to determine the implantation window of the endometrium. Other studies have worked in parallel on the endometrium and blood samples, finding similarities between miRNAs with expression linked to greater receptivity between both samples. An increase in miR-31 [160] or a decrease in miR-455-3p and miR-4423-3p [159] have been associated with the endometrial phase of greatest receptivity. Studies such as Di Pietro et al. (2018) [208] or Zhou et al. (2024) [209] in patients with chronic endometritis who present receptivity problems have shown elevated levels of miR-27a-3p and miR-124-3p in both endometrial tissue samples and serum. However, recent studies suggest that not only do miRNAs regulate the expression of genes important in endometrial receptivity but that there is cooperation between miRNAs and different isomiRs [210,211,212].

Even when the endometrium is most receptive, implantation failure can occur. Finding a cause has been the primary goal of studies on miRNAs implicated in implantation failure [213]. For the most part, these types of studies have recruited women with recurrent implantation failure (RIF), characterized by repeated embryo transfers without pregnancy (more than two attempts). Studies with this patient profile have been carried out in blood, endometrial tissue biopsies, and extracellular vesicles extracted from the endometrium. Zeng et al. (2021) [214] showed elevated levels of miR-6767-5p, miR-149-5p, and miR-4433b-5p and lower expression of miR-4511, miR-124-3p, miR-146-3p, miR-150-5p, miR-150-3p, miR-342-3p, and miR-874-3p in blood plasma and exosomes from it in women with RIF. Regarding extracellular vesicles originating from endometrial tissue, studies showed that miR-6131, miR-1246, and miR-218-5p were upregulated in women with RIF [163]. Among the upregulated miRNAs in endometrial tissue biopsies, miR-665 [162], miR-940, miR-144-3p [161], and miR-152-3p [159] stood out, while miR-20b-5p and miR-330-5p [161] were downregulated. However, there are discrepancies in miR-155-5p; for example, in the study by Chen et al. (2021) [161], its increase was associated with RIF, while in the study by Drissennek et al. (2022) [159], its levels were lower in the patient’s profile.

## 4. Challenges and Precautions in miRNA Work

As demonstrated throughout this review, miRNAs are of great importance not only for disorders related to fertility but also for many other pathologies. They have a crucial role in the pathophysiology of illnesses because they are determinants in gene silencing at the post-transcriptional level. Due to the increase or decrease in their levels associated with certain conditions, they could be biomarkers of these pathologies. The promise of miRNAs as future therapeutics was further explored by research demonstrating that their effects might be reversed by changing their levels, particularly through transfection experiments using mimics and inhibitors [215,216]. However, in many of the studies analyzed throughout this article, certain limitations have marked the potential of such studies. Therefore, some specific aspects must be considered in the study design to ensure robust results and less confusion due to avoidable biases.

### 4.1. Definition of Study Population

Given that infertility is associated with an increase in maternal age [217], recruitment in both groups must be age-matched if the research objective is focused on a reproductive outcome. In this way, there will be a guarantee that there are no statistically significant differences between the mean age in case and control groups. For example, the differences in AMH or AFC values between study groups were statistically significant in studies that refer to ovarian alterations. When checking the age of both groups, this variable was also different. Given that the AMH [218] value and the AFC [219,220] decrease as age increases, comparing two groups whose age variable differs between them could imply that the differences found are not due to the pathology but the aging process.

Similarly, the timing of the sample collection that will be studied during the research is crucial. Women have hormonal fluctuations throughout the menstrual cycle [221], and these changes directly affect the ovarian and uterine levels. Studies where sample collection is not unified, both in the ovary and the uterus, could lead to comparing different cycle phases and, thus, incorrect results. In those cases where sample collection is carried out throughout the fertility treatment, unifying the time of sample collection and the ovarian stimulation protocol is of great importance.

### 4.2. miRNA Techniques

Massive sequencing methods using Next-Generation Sequencing (NGS), microarray technology, and qPCR are the principal methods for studying miRNAs [222]. In the first and second cases, the costs are increased. Using NGS, it is possible to detect both the expression and sequence of all the miRNAs present in a sample. This technique requires the extraction of high-quality RNA and the preparation of miRNA libraries. Microarray technology is based on probes and is gradually being displaced by NGS. These two technologies are very useful in developing a large profile of the miRNAs differentially expressed between different groups. However, it is necessary to verify these results subsequently using qPCR [223], which is the gold standard to date. Given that many of the studies are based on techniques such as NGS and microarray, and later qPCR, it is important to divide the study population into two groups: the discovery population intended for these massive techniques and the subsequent validation population for qPCR. Both populations must comprise patients with the same profile but not the same subjects. This will guarantee that the results have been verified with two different techniques and allow the budget to be adjusted if the sample size of higher-cost techniques such as NGS or microarrays is reduced.

Regardless of the technique used, it is necessary to normalize the values obtained. Seyednasrollah et al. (2015) compared eight software packages and pipelines used in normalization for RNA sequencing and concluded that the tool used can affect the outcome of data analysis [224]. In the case of massive sequencing techniques, the decision will depend on the software incorporated into the technology used. However, in the case of qPCR, there is significant controversy among the studies since many use the U6 non-coding small nuclear RNA (RNU6). The use of this RNA as endogenous for cellular or tissue samples is widely accepted, although it does not have the exact nature and composition as a miRNA. But its use is not recommended when the type of study sample is blood, whether plasma or serum, and the objective is to analyze the expression of circulating miRNAs [225]. Given that RNU6 is part of the cell nuclear, its presence would indicate cell rupture in these cases. For this reason, it is suggested that during the initial screening techniques, NGS or microarray, miRNAs that do not show differential expression between the groups are also selected for subsequent use as endogenous miRNAs to perform normalization in the qPCR [226].

A final note regarding the use of blood samples, both plasma and serum, is the monitoring of hemolysis in the samples since it is a source of significant variability in the profile of expressed miRNAs [227]. This can be checked by measuring the absorbance of the sample at 414 nm, the absorbance of hemoglobin, or by calculating delta Cq [(miR-23a-3p)–(miR-451a)]. By assessing these two miRNAs, the level of hemolysis of the samples can be determined since the levels of miR-23a-3p are stable in serum and plasma and are not affected by hemolysis, and miR-451a is highly expressed in erythrocytes [228].

Another common mistake in working with miRNAs is failing to specify whether the differentially expressed miRNA is 5p or 3p [229]. During the maturation process of miRNAs, the passenger strand is degraded, leaving the functional one that will travel attached to the argonaute proteins to its target mRNA. Depending on the cell type or tissue, both strands may be functional or only one, and the target mRNAs may vary. For this reason, it is important to specify which strand is associated with the studied pathology.

### 4.3. miRNAs as Therapy

Once the alteration in the levels of a particular miRNA has been evident in a specific pathology and its effect on its target mRNA has been determined, it can be used as a biomarker of the disease. Subsequent studies with this miRNA will focus on the transfection of the interest cells with a mimic and/or inhibitor of that miRNA to explore its potential use as therapy [215,216]. In this type of study, it is necessary to consider two main aspects: (1) a miRNA can have multiple target genes and (2) preventing degradation of the miRNA until it reaches its target mRNA. Therefore, to reduce the effect of miRNAs on other tissues, the route of administration of miRNAs must be monitored for the use of therapies. If it does not work, try developing delivery or vehicle systems like viral vectors [217] or nanoparticles [203] that ensure the miRNA reaches the intended organ, which will prevent the breaking down in the blood and even reduce adverse immune reactions to miRNA.

## 5. Conclusions

Numerous studies have shown that miRNAs are crucial for post-transcriptional regulation, even if they do not code for proteins. Because of their ability to inhibit specific mRNA targets, they are molecules of tremendous interest for understanding pathophysiology and for usage as biomarkers or future treatments. Thanks to this scientific review article, we hope to provide clarity on the studies performed to date in women that relate miRNA profiles and reproductive alterations, thus establishing a solid foundation on which to build future research. In addition, conducting multiple meta-analyses on miRNAs and female infertility disorders could offer several benefits, such as helping to generalize the findings, increasing statistical power, identifying consistent patterns, improving reproducibility, reducing biases, and strengthening the conclusions.

## Figures and Tables

**Table 1:** miRNAs differentially expressed in ovarian disorders.

	DOR	POR	POI	PCOS	
**Upregulated**	miR-128-3p [89] miR-6881-3p [90] miR-484 [91] miR-4463 [92] miR-342-3p [93] miR-483-3p [93] miR-625-3p [93] * miR-28-3p [94] * miR-155-5p [94] miR-29a-5p [94]	* miR-23a [95] * miR-21-5p [96] miR-15a-5p [97]	* miR-23a [98] miR-27a [98]	miR-33b [99] miR-142 [99] miR-1298-5p [100] miR-212-3p [101] miR-490-5p [101] miR-4643 [101] miR-3131 [102] miR-206 [102] miR-204-5p [102] miR-100-5p [102] miR-193a-5p [102] miR-381-3p [103] miR-199b-5p [103] * miR-93 [104] -3p [103] miR-361-3p [103] miR-127-3p [103]	miR-1238-3p [103] miR-382-5p [103] miR-425-3p [103] * miR-222 [105] * miR-146a [105] -5p [106] miR-30c [105] * miR-21-5p [107] * miR-23a-3p [107] miR-26a-5p [107] * miR-223 [104] miR-126-3p [106] miR-151a-5p [108] miR-488 [108]
**Downregulated**	miR-221-3p [109] miR-16-5p [89] * miR-106a [110] miR-122-5p [93] miR-1246 [93] miR-130b-3p [93] * miR-21-5p [94]		miR-22-3p [111]	miR-423 [99] miR-647 [101] miR-539-5p [102] miR-650 [103] miR-663b [103] * miR-155 [112] miR-103-3p [107,113] miR-376a-3p [107,113] miR-19b-3p [107]	* miR-222-3p [107] miR-139-5p [113] * miR-28-3p [113] miR-320 [114] miR-20b-5p [106] * miR-106a-5p [106] miR-18a-3p [106] * miR-223-3p [108]

* indicates miRNAs that have been reported to be differentially expressed in several studies, either for the same or different conditions. More information can be found in Section 3.1.5. Differentially expressed miRNAs in follicular cells such as GCs or CCs are shown in blue. Those studied in FF appear in green, while those detected in blood (serum or plasma) are red. The miRNAs that combine both colors indicate that they have been detected in these two types of samples. DOR: diminished ovarian reserve; POR: poor ovarian response; POI: premature ovarian insufficiency; PCOS: polycystic ovarian syndrome.

**Table 2:** miRNAs differentially expressed in endometriosis and adenomyosis.

	Endometriosis		Adenomyosis
**Upregulated**	miR-30a [165] miR-93 [165] miR-325 [166] miR-492 [166] miR-520e [166] * miR-145 [167] miR-122 [168]	miR-199a [168] miR-125b-5p [169] miR-150-5p [169] miR-342-3p [169] miR-451a [169] * miR-30c-5p [170]	miR-17 [171] miR-191 [172] miR-181b [172] * miR-145 [173]
**Downregulated**	miR-143 [165] miR-203a-3p [166] miR-126-5p [174] miR-202-3p [175] miR-424-5p [175] miR-449b-3p [175] miR-556-3p [175] miR-199a-3p [176] miR-143-3p [176] miR-340-5p [176]	* let-7b [169,177] -5p [176] miR-21-5p [176] miR-103a-3p [176] * miR-17-5p [176,178] * miR-20a-5p [176,178] miR-22 [178] miR-31 [167] miR-135a [177] miR-3613-5p [169]	miR-10b [172] miR-200c [172] let-7a [179] * miR-30c-5p [180] miR-183 [181]

* indicates miRNAs that have been reported to be differentially expressed in several studies, either for the same or different conditions. Differentially expressed miRNAs in endometrial tissue are shown in grey. Those studied in blood (serum or plasma) appear in red.
